# A Multi-modelling Approach for Assessing Sustainable Tourism

**DOI:** 10.1007/s11205-022-02943-4

**Published:** 2022-05-28

**Authors:** Gennaro Punzo, Mariapina Trunfio, Rosalia Castellano, Mirko Buonocore

**Affiliations:** 1 Department of Economic and Legal Studies, University of Naples “Parthenope”, Via Generale Parisi, 13, 80132 Naples, Italy; 2 Department of Management and Quantitative Studies, University of Naples “Parthenope”, Via Generale Parisi, 13, 80132 Naples, Italy

**Keywords:** Sustainable tourism, Composite indicator, Multi-modelling approach, Assessment, Italian regions

## Abstract

Academics, institutions and policymakers advocate systematic assessments to design sustainable development and implement proper environmental management; however, practical measurements in tourism research based on composite indicators are still in progress. This paper aims to build and validate a composite indicator of sustainable tourism (SusTour-Index), which recognises the economic, environmental and social dimensions as the three main interrelated facets of tourism sustainability. The SusTour-Index is composed of 75 elementary indicators, adequately structured in pillars and sub-pillars within each economic (34), environmental (21) and social dimension (20). A multi-modelling approach tests the hierarchical structure of the SusTour-Index by combining different weighting and aggregation methods within each sustainability dimension to choose the most appropriate model once the uncertainty analysis has been performed. The structure of the SusTour-Index is validated in all 21 Italian regions by performing 23 different models of the same composite indicator. The paper presents theoretical and methodological contributions for future research and advances in practical assessments, supporting policymakers and institutions in planning and managing sustainable tourism development.

## Introduction

For several years, academics, policymakers, and international organisations have required systematic assessments to design sustainable development and implement proper environmental management that combines economic growth with social and natural capital protection. In this context, sustainable tourism development has emerged as the dominant paradigm to redress the cumulative negative impacts of tourism development (Bramwell et al., 2017; Pagliara et al., 2021; Ruhanen et al., 2015; Zhong et al., 2011).

Sustainable tourism is firmly positioned in the United Nations 2030 Agenda for Sustainable Development Goals (SDGs) and potentially involves, directly or indirectly, all the goals. In particular, the Agenda includes, among other things, these targets: (1) implementing policies to promote sustainable tourism that creates jobs and encourages local culture and products (target 8.9); (2) developing tools to monitor sustainable development impacts for sustainable tourism (target 12.b); (3) increasing economic benefits for the sustainable use of marine resources, including through sustainable management of fisheries, aquaculture and tourism (target 14.7); (4) preserving biodiversity and ecosystems through sustainable tourism that helps reduce waste and consumption (goal 15). Therefore, it is desirable to provide practical advances in tourism sustainability measurement that help policymakers verify existing tools or design coherent new policies of sustainable tourism development towards the SDGs. In this field, monitoring tourism development through a broad set of indicators can be strategically important to combine community needs with the sustainable management of natural heritage and cultural resources.

A strand of literature has recognised sustainability indicators as a solid methodology and has proposed different types of indicators (mainly descriptive and related to specific destinations or geographical areas). Scholars have also renowned the need for composite indicator methodologies to design proper tourism planning and management practices and actions (Arbolino et al., 2021; Asmelash & Kumar, 2019; Choi & Sirakaya, 2006). Butler's seminal contribution (1999) argued that sustainability is 'meaningless' without indicators. Likewise, international organisations have accepted composite indicators measuring tourism impacts and sustainable development as fundamental tools for monitoring strategic policies towards sustainable tourism (UNWTO, 2004) and for communicating to society and destination stakeholders. However, practical assessments in tourism research based on composite indicators are still ongoing (Blancas et al., 2016; OECD, 2016; Torres-Delgado & Saarinen, 2014).

This paper helps fill this research gap by building and validating a composite indicator of sustainable tourism, the Sustainable Tourism Index (SusTour-Index), which recognises the economic, environmental and social dimensions as the three main interrelated facets of tourism sustainability. This study performs a multi-modelling approach to identify the most suitable methodology to summarise the elementary indicators according to the hierarchical structure of the SusTour-Index. Different combinations of weighting and aggregation methods are tested, resulting in alternative models of the SusTour-Index within the same theoretical framework (OECD, 2008; Saisana et al., 2011). The hierarchical structure of the SusTour-Index is validated in all 21 Italian regions at the NUTS-2 level, using a large set of elementary indicators from official statistical sources.^1^ Italy was chosen as one of the most significant worldwide destinations (5th for tourist arrivals by UNWTO, 2018) in light of the specific strengths and weaknesses of the tourism sector and the overall implications in terms of sustainability (Castellano et al., 2019). Besides, Italian tourism policies are defined and managed at the regional level.

The paper presents theoretical and methodological contributions, opening rooms for future research in the practical assessment of economic, environmental and social impacts, and supports policymakers in planning and managing sustainable tourism development.

## Sustainable Tourism Composite Indicators: A Review

### The Role of International Institutions

Since the 1990s, international institutions have recognised sustainability indicators as relevant tools for efficient destination policy making and for planning and management processes, providing an integrated information system to assess the impact of tourism, meant as *economic* activity, on the *environment* and *society* (UNWTO, 1996, 2004, 2005). Over the years, authoritative international institutions—e.g. World Tourism Organization of the United Nations (UNWTO), European Commission (EC), European Environment Agency (EEA), and Organization for Economic Cooperation and Development (OECD)—have developed theoretical frameworks and methodologies for composite indicators of sustainable tourism, often based on sustainable development standards.

The Pressure-State-Response (PSR) model proposed by OECD (1994) and its extended version of the Driver-Pressure-State-Impact-Response (DPSIR) model by EEA (1999) represents the seminal analytical frameworks to analyse the interactions between human behaviour and the environment. Despite being more focused on environmental aspects, the PSR and DPSIR models are considered reference points for scholars when selecting relevant indicators in every research field, trying to classify each indicator according to the element of the DPSIR model.

The DPSIR model allows for analysing the impact of human activities on the environment and society's response to these changes. The DPSIR approach includes indicators able to describe the specific sector itself and the related economic and policy considerations placed in the context of sustainable development. The interaction between tourism and the environment are identified by indicators to be developed for each dimension of the DPSIR model. The connection system provided by the DPSIR model requires the initial establishment of the causal relationship between the elementary indicators and the dimensions to which they belong. This is not a simple task for tourism indicators. The impact of tourism cannot be defined a priori as a cause or effect because of its heterogeneity (e.g. geographical dimension, type of tourism, seasonality). Therefore, the DPSIR model is suitable for studies on sustainable tourism aiming at investigating the impact of tourism on the environment, but a comprehensive approach is rather complicated.

UNWTO (2004) provided indicators for sustainable tourism measurement, identifying more than 150 sub-components and 768 elementary indicators developed by a wide range of experts. Moreover, the common issues of destinations are determined in twelve core indicators, labelled 'basic indicators', and included in a list of 'baseline indicators'. They can be customised according to the territorial level of analysis and enriched with other indicators to focus on the peculiarities of each specific destination (e.g., coastal, maritime, mountain).

The European Commission promoted tourism sustainability quite late by developing the European Tourism Indicators System (ETIS), a toolkit of sustainable destination management, presenting four fundamental dimensions of tourism sustainability (European Commission, 2016): environmental impact, social and cultural impact, economic value, and destination management.

### Sustainable Tourism Composite Indicators in the Literature

Considerable progress has emerged in academic research on sustainable tourism (Ruhanen et al., 2015), moving beyond the myopic view of the environmental perspective towards a holistic approach, which also includes economic and social dimensions and political/institutional aspects (Choi & Sirakaya, 2006; Ko, 2005). An intense debate was therefore aimed at effectively supporting decision-makers in achieving the sustainable development goals through indicators capable of providing an integrated information system to assess the impact of tourism (UNWTO, 2004, 2005).

An indicator-based approach to measuring tourism sustainability is a growing research topic, but many composite indicators remain theoretical conceptualisations with marginal empirical implementation (Blancas et al., 2016; Lee & Hsieh, 2016; Ruhanen et al., 2015). Analyses mainly focus on single tourism destinations (e.g. region, island, park) and use different pillars and indicators (local approach), which compromise their comparability at the regional and national level (Chris & Sirakaya, 2006; McCool et al., 2001; Torres-Delgado & Saarinen, 2014). Currently, scholars call for developing a more organic system of indicators for assessing tourism development and impact (Budeanu et al., 2016).

Table 1 summarises the main composite indicators of sustainable tourism proposed by the literature, explaining concepts (dimensions and innovativeness), elementary indicators (economic, environmental, social and others), methodology (approaches, weighting, aggregation), advantages and disadvantages.

**Table 1:** Sustainable tourism composite indicators in the literature

Authors	Concept of sustainable tourism indicator	Investigation area	Economic (elementary indicators)	Environmental (elementary indicators)	Social (elementary indicators)	Other dimensions (elementary indicators)	Statistical Methodology	Advantages and disadvantages
Asmelash and Kumar (2019)	Four dimensions of Sustainable Tourism Development: (1) Socio-cultural sustainability (2) Environmental sustainability (3) Economic sustainability (4) Institutional Sustainability Each dimension is a set of further sub-dimensions	Tigrai Regional State (Ethiopia)	11 elementary indicators structured in 3 sub-dimensions: Employment quality (4) Economic viability (3) Local prosperity (4)	12 elementary indicators ordered into 4 sub-dimensions: Physical integrity (3) Biological diversity (3) Resources efficiency (3) Environmental purity (3)	12 elementary indicators organised into 5 sub-dimensions: Social equity (3) Visitor fulfillment (4) Local control (4) Community wellbeing (4) Cultural richness (3)	Institutional dimension It is composed of 18 elementary indicators, which in turn are structured in 4 sub-dimensions: Local-oriented control policy (3) Political participation (3) Local policy planning (3) Political support (3)	Three-round Delphi Method to finalize the evaluation of the potential indicators Exploratory Factor Analysis (EFA) and Structual Equation Modelling (SEM) were performed to uncover the underlying structure of the relatively large set of sustainability indicators	Advantages: Broad-based involvement of stakeholders in indicator development The use of EEA’s DPSIR comprehensive framework The inclusion of the institutional sustainability Disadvantages: Sustainable indicators are strictly dependend on the specific local destination, which makes it hard to be set at the regional and national level Elementary indicators based on the participatory approach are too limited to the given tourist sites The use of a convenient sampling method in the pilot study makes it hard to infer the analysis, which would have been much more adequate with probability sampling techniques
Blancas et al. (2010a)	*Net Goal Programming Synthetic Indicator (GPSI*^*N*^*)* Three dimensions of Sustainable Tourism Development: (1) Socio-cultural sustainability (2) Environmental sustainability (3) Economic sustainability Each dimension shows a hierarchical structure	14 coastal countries of Andalusia (Spain)	34 elementary indicators organised into 8 key aspects: Economic benefits for tourism for the local population and destination (11) Sustaining tourist satisfaction (3) Development control (1) Tourist offers providing a variety of experiences (6) Seasonality (3) Tourism employement (2) Transport related to tourism (6) Competitiveness of the destination (2)	32 elementary indicators organised into 8 key aspects: Protection of the natural resources (2) Management of scarce natural resources (5) Waste management and treatment (9) Atmospheric pollution (3) Management of the visual impact of infrastructure (4) Use intensity (4) Environmental management (1) Specific aspects of environmental tourism sustainability (4)	28 elementary indicators organised into 6 key aspects: Social-cultural effects of tourist activity (7) Conservation of cultural heritage (3) Destination safety (2) Control for the effects of tourist development on the population (9) Social-carrying capacity (2) Effects of tourist activity on the host population’s well-being (5)	−	GPSI^N^ is based on the goal programming method (from Operation Research) under which the destination is compared, for each indicator, to a priori aspiration level (given by a panel of experts according to the opinion-based approach) GPSI^N^ is the weighted sum of two components, which synthesise the strenghtness and weakness of each destination	Advantages: The composite indicator building avoids previous normalisation The compensation in GPSI.^N^ depends on two parameters, λ and γ, which are relative weights of strengths and weaknesses, respectively GPSI.^N^ is open-ended measure to the goals of sustainable tourism Two phases of data aggregation: the first one produces a synthetic indicator for each dimension; the last provides a final rank to analyse its stability Disadvantages: Subjectivity of expert panel to define the a priori aspiration level and weights of each indicator The adaptability of indicators to goal programming may have some conflicts between areas with different tourism targets
Blancas et al. (2010b)	Three dimensions of Sustainable Tourism Development: (1) Social (2) Environmental (3) Economic The dimensions do not show any structure inside (for a total of 32 elementary indicators)	32 coastal areas in Spain (Spain)	8 elementary indicators	16 elementary indicators	8 elementary indicators	−	The key factors in the indicators system are relevance, data availability, spatial scope and feasibility of performing comparative Data-centric approach based on two-stage aggregation methodology by PCA and a distance to a reference point (DPC)	Advantages: Specificity on local site (coastal tourism) Objective weights by DPC Disadvantages: A not full comprehensive system of indicators A low communicative and interpretability of results
Blancas et al., (2016)	*Vectorial Dynamic Composite Indicator* (VDCI) An innovative composite indicator, which is a Vector with a Static (SC) and Dynamic Component (DC) VDCI is structured in three dimensions: (1)Social(2) Economic (3) Environmental	29 European countries	36 elementary indicators organised into 8 key aspects: Economic benefits of tourism for the host community and destination (10) Sustaining tourist satisfaction (2) Development control (1) Tourist offers: providing a variety of experiences (5) Seasonality (3) Tourism employement (7) Tourism-related transport (7) Destination competitiveness (1)	20 elementary indicators organised in 10 key aspects: Protection of the natural ecosystem (1) Energy management (3) Water management (1) Wastewater management (2) Management of solid urban waste (4) Atmospheric pollution (3) Management of the visual impact of facilities and infrastructure (3) Intensity of tourist use (1) Public administrations’ expenditure on environmental protection (1) Use of resources (1)	29 elementary indicators organised into 6 key aspects: Socio-cultural effects of tourist activity (4) Safety of destination (5) Conservation of cultural heritage (2) Effects on national population structure (6) Social carrying capacity (2) Effects on level of well-being in the local population (10)	−	VDCI is applied in the Static Component (SC) to evaluate the destination level in relation to other competiting destinations, in order to reach a common aspiration level The Dynamic Component (DC) allows the analysis in the reference period of the progress−regress of each destination respect to the own aspiration level	Advantages: Comparative analysis between destinations, and over-time analysis for each destination Adaptability to other tourism destinations by incorporating new specific indicators Direct analysis of the strengths and weaknesses of each destination respect to the three dimensions Disadvantages: Need to define a set of shared goals for each destination to evaluate the dynamic component ranking, as well as a common reference value for the static component ranking
Blancas et al. (2018) Lozano-Oyola et al., (2019a)	*Differential Dynamic Index* (DDI) with a Static (SC) and Dynamic Component (DC) DDI modified the previous VDCI (Blancas et al., 2016) to obtain differentiated vectorial sustainability evaluations for each type of territory using multiple benchmarks DDI is composed of the same three dimensions of VDCI with baseline aspects Lozano-Oyola (2019a) used the analytic information provided by the DDI’s two components for the creation of a System of Sustainable Tags to link the evaluation of the indicator with the planning and management decisions of the destinations	Coastal destinations of Andalusian (Spain)	24 elementary indicators structured into 8 baseline aspects: Economic benefit of tourism for the host community and destination (6) Sustaining tourist satisfaction (2) Development control (1) Tourism facilities on offer-provision of a variety of experiences (7) Seasonability of tourism activity (3) Tourism employment (2) Tourism-related impact transport (2) Destination competitiveness (1)	20 elementary indicators structured into 9 baseline aspects: Protection of the natural ecosystem (2) Energy management (2) Water management (1) Wastewater management (2) Management of solid urban waste (5) Atmospheric pollution (3) Management of the visual impact of facilities and infrastructure (3) Intensity of tourist use (1) Environmental management (1)	21 elementary indicators structured into 6 baseline aspects: Socio-cultural effects of tourism on host community (4) Local public safety (2) Conservation of cultural heritage (3) Effect on local population structure (6) Social carrying capacity of the destination (2) Effects on level of well-being in the local population (4)	−	A comprehensive set of 65 elementary indicators Weighting system based on a panel of 57 experts (Budget Allocation Process) with three weighting levels A cluster analysis to form homogeneous groups of municipalities	Advantages: Different aspiration levels according to each territory’s characteristics Using the Sustainable Tourism Evaluation Diagram to evaluate both the current situation of each destination and its progress or regress over time Disadvantages: It would require other relative dynamic measures that enable the quantitative grading of the destination’s evaluation according to the evolution shown by a reference territory
Castellani and Sala (2010)	*Sustainable Performance Index* (SPI) SPI summerises 20 elementary indicators concerning economic, social, cultural and environmental aspects without any over structure	Alpi Lepontine Mountains Community -union of 13 municipalities (Italy)	Economy and labour (6)	Environment (5)	Population (3) Housing (1)	Tourism (2) Services (3)	A comprehensive set of 20 elementary indicators is inspired by European projects (DIAMONT, MARS) The framework includes subjective information by local stakeholders to promote local sustainable development (following the European Charter methodology) The weighting of each elementary indicator is obtained by objective and subjective values	Advantages: Linkage to local policy targets Repeatability and comparability of procedure among mountain protected areas Disadvantages: The subjectivity of carrying capacity’s threshold The strong dependence on the local situation, which makes it hard to be set at the regional and national level
Lozano-Oyolaet al. (2019b)	Three dimensions of Sustainable Tourism: Social (1) Economic (2) Environmental Each dimension mostly includes objective indicators (for a total of 49 elementary indicators), without showing any structure inside	36 Andalusian Urban Destinations (Spain)	20 elementary indicators	15 elementary indicators	14 elementary indicators	−	Weighting system based on expert panel (Budget Allocation Process) with three levels: dimensional, factorial and quantification Non-compensatory aggregation procedure based on multicriteria decision-making ideas and on the construction of a mixed-integer linear programming model	Advantages: It allows obtaining a complete pre-order of alternatives Disadvantages: Computational cost required for determining the ranking
Pérez et al. (2013)	DEAPC index (*Data Envelopment Analyis after distance-Principal Component*) Three dimensions of Sustainable Tourism Development: (1)Social dimension related to people and tourist development (2) Economic dimension related to tourism management, and material and financial resources (3)Patrimonial dimension related to natural and cultural environment Each dimension includes both subjective and objective indicators (for a total of 39 elementary indicators), without showing any structure inside	15 Cuban nature-based tourism destinations (Cuba)	14 elementary indicators	Some indicators of tourism environmental sustainability are included in the patrimonial dimension	11 elementary indicators	Patrimonial dimension which is composed of 14 elementary indicators	A two-stage methodology (Data Envelopment Analysis after distance-Principal Component – DEAPC): (1)Distance-Principal Component (DPC), which combines Principal Component Analysis (PCA) with the distance to a reference point (dimensional composite indicators) (2)Benefit-of-the-Doubt approach (BoD) for the global synthetic indicator	Advantages: Mixture of objective (obtained from statistical sources) and subjective (reflecting the perceptions of all agents involved in tourism development) indicators The use of the concept of sustainable tourism development proposed by WTO (2004) The combination of the more objective statistical methods with efficiency methods The possibility to be applied to other destinations by changing some elementary indicators Disadvantages: The presence of the hybrid patrimonial dimension, which includes both cultural aspects and more strictly environmental aspects that could be form a separate dimension Indicators are strictly dependend on the specific local destination, which makes it hard to be set at the regional and national level
Pulido Fernández and Sánchez Rivero (2009)	*Sustainable Tourism Index* (STI) The global STI method summerises 14 elementary indicators by the Spanish System of Environmental Indicators of Tourism, refusing the hypothesis that all indicators are equally important	17 Spanish autonomous regions (Spain)	\	\	\	\	The STI method uses a rubust weighting system based on factor loadings A comparison of STI method with other two aggregation methods, which gained worldwide acceptance: Tourism competitiveness monitor of the World Travel and Tourism Council (WTTC) Environmental Sustainability Index (ESI) of the World Economic Forum (WEF) The comparison is based on composite correlations among the indicators built with the three methodologies using the Spanish system of 14 environmental tourism indicators (SSETI)	Advantages: Consistency of weighting system The use of EEA’s DPSIR comprehensive framework Disadvantages: Four partial indexes of sustainability corresponding to each component of the DPSIR model The paucity of data makes it hard to extend the theoretical framework to different tourist destinations, compromising their comparability
Torres-Delgado and Palomeque, (2018)	*Index of Tourist Sustainability* (ISOST) Three dimensions of Tourism Sustainability: (1)Socio-cultural dimension (2) Economic dimension (3) Environmental dimension From an initial list of 26 elementary indicators – taken from the indicator system at the local level developed by Torres-Delgado and Lόpez Palomeque (2014) – to a final list of 12 indicators	20 tourist municipalities in Catalonia (Spain)	3 elementary indicators: Seasonality of tourism offer Presence of second homes Public investment in tourism	6 elementary indicators: Energy consumption Water consumption Waste generation Land use distribution Environmentally certified tourism establishments Environmental criteria applied to tourism planning	3 elementary indicators: Tourist population Diversification of tourist attractions and resources Tourism products accessible to disabled		OECD (2008) methodology: *z*-score method Equal weight system Linear aggregation at both levels (elementary indicators and sub-indexes)	Advantages: The use of EEA’s DPSIR comprehensive framework: within each dimension of tourism sustainability, the elementary indicators are declined with respect to each component of the DPSIR model The high level of territorial detail (tourist municipalities) allows sustainable tourism policies to be most effective Disadvantages: Linear aggregation could imply compensability, i.e., poor performance in some elementary indicators can be compensated by high values of other indicators

The composite indicators of sustainable tourism mainly focus on three typologies of elementary indicators: economic, environmental and social (Blancas et al., 2010a, 2010b; Bonett, & Wright, 2015; Lozano-Oyola et al., 2019a, 2019b; Torres-Delgado & Lόpez Palomeque, 2018). Some scholars considered the institutional (Asmelash & Kumar, 2019) and patrimonial (Pérez et al., 2013) dimensions and integrated the elementary indicators of tourism and services in the framework of sustainable tourism (Castellani & Sala, 2010).

From a methodological perspective, several authors adopted the EEA’s DPSIR model (Asmelash & Kumar, 2019; Castellani & Sala, 2010; Pulido Fernández & Sánchez Rivero, 2009; Torres-Delgado & Lόpez Palomeque, 2018). Composite indicators were also built on expert opinions or participatory processes (Asmelash & Kumar, 2019; Blancas et al., 2010a). Several methodologies have been applied, which have both advantages and disadvantages; however, most proposed composite indicators suffer from the lack of consensus on the best methodology for their construction and validation (Torres-Delgado & Lόpez Palomeque, 2018).

Therefore, building a system of sustainable tourism indicators that is scientifically advanced, methodological solid and useful for decision-making represents the current challenge for scholars (Blancas et al., 2016; Tanguay et al., 2013) with a variety of implications.

First, sustainability's multidimensionality makes it hard to aggregate a considerable amount of information (Butler, 1999; Castellani & Sala, 2010; Miller, 2001) and identify the universal list of elementary indicators. UNWTO (2004) has identified more than 150 sub-components and 768 sustainable indicators with twelve baselines that can be customised according to the territorial level of analysis and destination characteristics (e.g. coastal, maritime, mountain). Furthermore, both academics and international institutions have proposed selection criteria for indicators to improve comparability and reliability at the different geographical levels required (European Commission, 2016; Tanguay et al., 2013).

Second, once the hierarchical structure has been identified, the choice of the appropriate methodology (i.e. for weighting and aggregating elementary indicators and pillars/sub-pillars) can be affected by subjective judgments, which reflect on the outcome of the composite indicator (e.g. Mayer, 2008; Singh et al., 2009; Torres-Delgado & Lόpez Palomeque, 2018).

Although diverse research fields have applied the multi-modelling as a methodology to gather and summarise the information contained in a set of elementary indicators (Castellano & Rocca, 2016, 2019; Saisana et al., 2011, 2020), it is still almost unexplored in the field of tourism sustainability (Mikulić et al., 2015).

## Material and Methods

This study builds and validates a composite indicator of tourism sustainability (SusTour-Index), which summarises the multidimensionality of sustainable tourism in the economic, environmental and social dimensions. The research design considered both the guidelines of international institutions (EC, 2016; EEA, 1999, 2003; OECD, 1994; UNWTO, 2004, 2005) and the literature's milestones on composite indicators of sustainable tourism (Table 1).

The multi-modelling approach combines a comprehensive spectrum of methodological choices, which generate a plurality of SusTour-Index models coherently with the underlying theoretical framework. While most studies considered one specific methodology for building a composite indicator of sustainable tourism, we obtain as many SusTour-Index models as combinations of weighting and aggregation methods within each dimension of sustainability. This made it possible to determine the effect of varying methodological assumptions in treating the indicators on the outcomes (scores and rankings) of the SusTour-Index, guiding the choice of the most suitable methodology once the uncertainty analysis has been performed (Saisana et al., 2011).

The SusTour-Index was structured following the OECD (2008) guidelines, which outline an ideal sequence of steps for building rigorous composite indicators: (1) developing the theoretical framework, (2) selecting elementary indicators, (3) treatment of missing data, (4) multivariate analysis, (5) data normalisation, (6) weighting and aggregation, (7) uncertainty analysis. The SusTour-Index was designed with maximum transparency in each step which allowed us to retrace step-by-step the composite indicator's construction.

### Developing the Theoretical Framework and Selecting Elementary Indicators

**Fig. 1:**
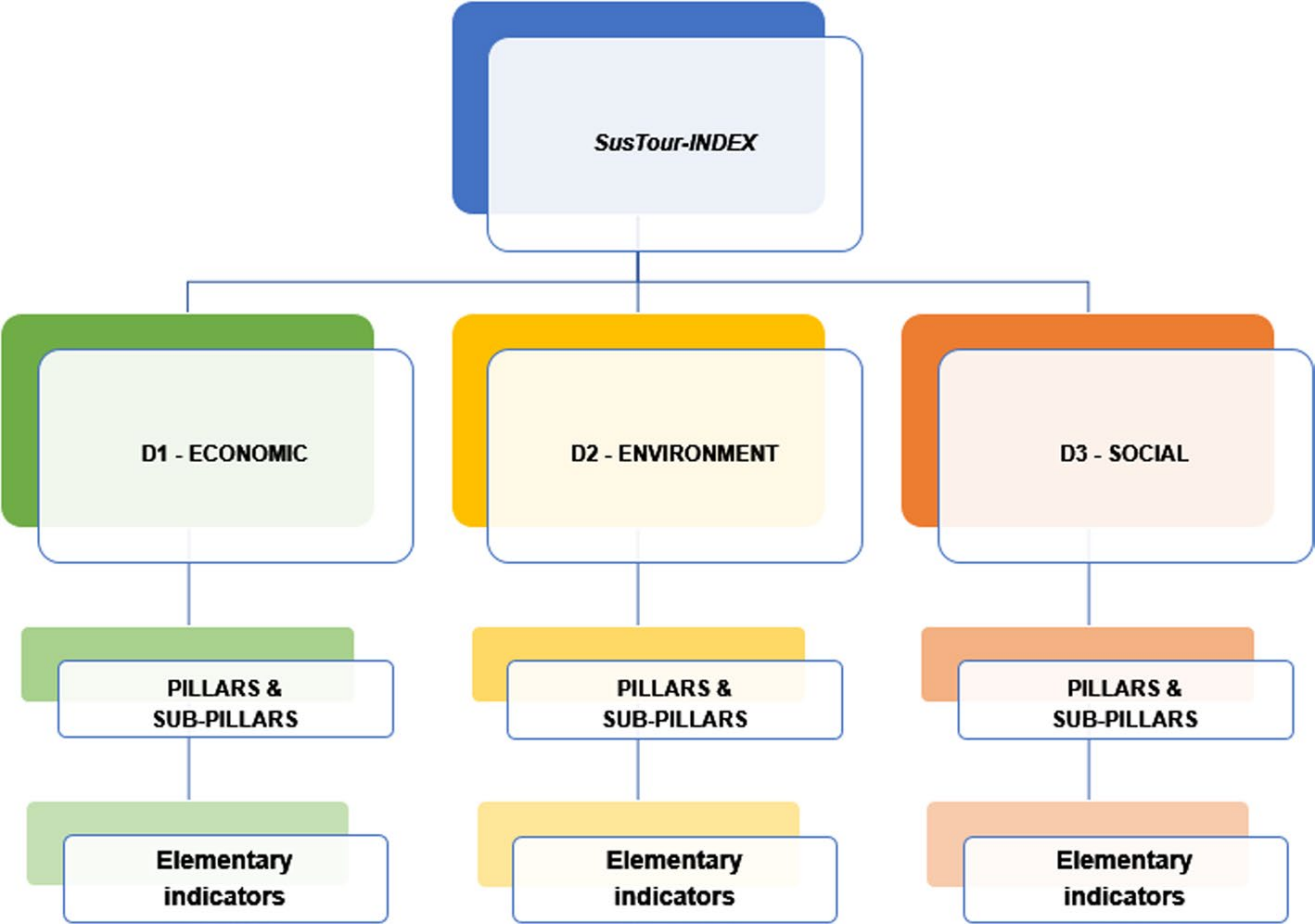
The hierarchical structure of the SusTour-Index

The first two steps of the OECD procedure defined the conceptual structure of the SusTour-Index and the elementary indicators that give the best evidence of each dimension's meaning (sub-index) of tourism sustainability according to the hierarchical architecture in pillars and sub-pillars (Fig. 1; Tables 2, 3, 4).

**Table 2:** Economic dimension of the SusTour−Index

Pillar	Sub-Pillar	Ind. #	Indicator	Description	Source
Economic value (D1.1)		ind.01	GDP generated by tourism	Share of GDP generated by tourism out of total regional GDP	Istat
		ind.02	Labour productivity in the tourism sector	Value added by the tourism sector per work unit in the sector – thousands of euro chained together	Istat
		ind.03	Tourism intensity	Share of tourists out of inhabitants	Ispra
		ind.04	Summer seasonality of tourism	Days of presence (Italian and foreign) in the complex of accommodation facilities in the non-summer months per inhabitant	Istat
		ind.05	Employment in the tourism sector	Percentage of employees in the tourism sector	Istat
		ind.06	Female employment in tourism	Percentage of female employees in the tourism sector	Istat
		ind.07	At-risk-of-poverty persons	Percentage of at-risk-of-poverty persons with an equivalent income to or below 60% of the median equivalised income in the regional resident population	Istat-BES
Sustainability of tourism enterprise (D1.2)		ind.08	Tourism companies	No. of tourism companies (in 1 K); Istat survey on the Capacity and occupancy of tourist accommodation establishments	Istat
		ind.09	Accommodation establishments	No. of accommodation establishment (hotels, camping, B&B, …); Istat survey on the Capacity and occupancy of tourist accommodation establishments	Istat
		ind.10	ISO 14001 certification	No. of ISO 14001 certified production sites	Ispra
		ind.11	EMAS registration	No. of EMAS (Eco-Management and Audit Scheme) registered sites	Ispra
		ind.12	Ecolabel license	No. of EU Ecolabel licences between services and products	Ispra
International appeal (D1.3)	International tourism demand (D1.3.1)	ind.13	Tourist stay of foreigners	No. of foreign overnight stays	Bank of Italy
		ind.14	Quota of foreign tourism on domestic tourism	Ratio of no. of foreign arrivals to Italian arrivals	Eurostat
		ind.15	Foreign tourism expenditure	Total foreign tourism expenditure (in mln. euro)	Bank of Italy
		ind.16	Foreign tourists revisiting a place	No. of foreign tourists revisiting a place	Bank of Italy
	International tourism satisfaction (D1.3.2)	ind.17	Courtesy of the host population for foreign tourism	Rating on 5-points Likert scale of the courtesy of the host population for foreign tourists	Bank of Italy
		ind.18	Cooking for foreign tourism	Rating on 5-points Likert scale of local cooking for foreign tourists	Bank of Italy
		ind.19	Environment for foreign tourism	Rating on 5-points Likert scale of the quality of the environment visited by foreign tourists	Bankof Italy
		ind.20	Value of visited cities for foreign tourism	Overall rating on 5-points Likert scale of the cities visited by foreign tourists	Bank of Italy
Tourism demand (D1.4)		ind.21	Tourism arrivals	No. of tourist arrivals (Italian and foreign)	Istat
		ind.22	Tourism presence in accommodation establishments	No. of presence of tourists (Italian and foreign) in accommodation facilities	Istat
		ind.23	Tourist stay	Average tourist stay	Istat
		ind.24	Ratio of tourist presence to resident population	Ratio of tourist visits to regional resident population	Istat
		ind.25	Museum visitors	No. of visitors to museums and similar institutions (in 100 K)	Istat
		ind.26	Cultural heritage visitors	No. of visitors to antiques and art institutes (thousands)	Istat
Cultural heritage organisations (D1.5)		ind.27	UNESCO world heritage	UNESCO world heritage in each Italian region	Unesco
		ind.28	UNESCO world heritage nomination	UNESCO world heritage nomination	Unesco
		ind.29	Archaeological heritage	No. of archaeological sites; Istat survey on museums and similar institutions	Istat
		ind.30	Museum heritage	No. of museums and art galleries; Survey on museums and similar institutions	Istat
		ind.31	Monumental sites	No. of monumental sites; Survey on museums and similar institutions	Istat
		ind.32	Degree of promotion of cultural offer of state institutes	Ratio of paying visitors to non-paying visitors of the state institutions of antiques and art with admission fee (%); Survey on museums and similar institutions	Istat
		ind.33	Cultural events	No. of theatrical and musical shows	Istat-SIAE
		ind.34	Expenditure for cultural events	Spending at the box office for theatrical and musical events	Istat-SIAE

**Table 3:** Environmental dimension of the SusTour−Index

Pillar	Sub-Pillar	Ind. #	Indicator	Description	Source
Energy and water consumption (D2.1)		ind.35	Energy consumption by inhabitants	Per capita energy consumption in KWh (elaborated by Istat on Terna Spa data)	Istat
		ind.36	Energy consumptio by tourism sector	Energy consumption by tourism enterprises (hotels, camping, restaurants,…) in mln KWh; Istat on Terna Spa data	Istat
		ind.37	Water consumption by inhabitants	Per capita water consumption (m.^3^)	Istat
		ind.38	Air pollution	No. of days the particulate threshold value is exceeded 50 ml/PM_10_	Istat-Ispra
		ind.39	Greenhouse gas (GHG) emissions by tourism industry	Share of CO_2_ (tonnes) by tourism industry out of total CO_2_, related to the land use and excluding emissions by maritime traffic	Istat
Sustainable energy and water management (D2.2)		ind.40	Energy consumption by renewable sources	Electricity from renewable sources (wind, biomass, solar heat, hydroelectric) as a percentage of gross internal consumption of electricity (GWh); Istat on Terna Spa data	Istat
		ind.41	Renewable energy produced as a percentage of the total	Ratio of energy from renewable sources (excluding transport sector) to total final energy consumption; Istat on Terna Spa data	Istat-ASVIS
		ind.42	Population served by water purification	Percentage of population actually served by urban waste water treatment plants with secondary and tertiary treatment over the total population of the region	Istat-ASVIS
		ind.43	Water distribution efficiency	Percentage of the volume of water supplied to users compared to that fed into the water system	Istat-ASVIS
Waste management (D2.3)		ind.44	Waste produced by tourism sector	Amount of urban waste attributable to tourism per capita (elaborated by ISPRA on Istat data)	Istat-Ispra
		ind.45	Rate of landfill waste to the total waste	Urban waste going to landfill as a percentage of the total urban waste collected	Istat-ASVIS
		ind.46	Urban waste collected separately	Tons of urban waste collected separately out of the total urban waste collected	Istat-ASVIS
		ind.47	Recycled rate	Ratio of the quantities of urban waste prepared for re-use or recycled to the total quantities produced	Istat-ASVIS
		ind.48	Per capita separate waste	Quantity of urban waste collected separately per capita (kg/inhabitant)	Ispra
		ind.49	Rate of organic waste	Organic fraction treated in composting plants out of total urban organic waste (%)	Istat-Ispra
Environmental value (D2.4)	Quality of the environment (D2.4.1)	ind.50	Blue Flag beach certification	No. of Blue flag beach or certified municipalities	FEE*
		ind.51	Favourable habitat conservation	Percentage of habitats with favourable conservation status	Istat
		ind.52	Quality of bathing coasts	Percentage of the length of the bathing coasts on the total length of the coasts	Istat-Ispra
	Promotion of natural heritage (D2.4.2)	ind.53	Sites of Community Importance (SCI)	Area of sites of community importance on the regional territory (%)	Istat-BES
		ind.54	Special Protection Area (SPA)	Percentage of official protected natural areas on the total territory	Istat-BES
		ind.55	Rete Natura 2000	Area of the Rete Natura 2000 (sites of community importance) on the regional area (%)	Istat-BES

*Foundation for Environmental Education

**Table 4:** Social dimension of the SusTour−Index

Pillar	Sub-Pillar	Ind. #	Indicator	Description	Source
Security (D3.1)		ind.56	Thefts and robberies suffered by tourists	No. of thefts and robberies potentially suffered by tourists	Istat
		ind.57	Percentage of crimes suffered by tourists to the total crimes	Ratio of no. of thefts and robberies suffered by tourists to the total crimes reported to law enforcement agencies	Istat
		ind.58	Petty crime index	Petty crime in the provinces of the regional capitals on the resident population	Istat
		ind.59	Road accident rate	Road accident rate per houndred inhabitants	Istat
		ind.60	Severe injury rate from road accident	Rate of serious injury in road traffic accident by region per 100 K inhabitants (hospital discharge data)	Istat
		ind.61	Occupational accident rate in the tourism sector	Accident rate in the tourism sector by number of employees	Istat-Inail
Health (D3.2)		ind.62	Mortality rate	Standardised mortality rate for major causes of death in 30–69 years per 100 K inhabitants	Istat
		ind.63	Hospitals	No. of hospitals and treatment centers per 100 K inhabitants	Istat
		ind.64	Family health care expenditure	Health expenditure per capita (including spending for all privately and publicly funded family health care services and products)	Istat; HFA-Italy (Health for All)
Mobility (D3.3)		ind.65	Local Public Transport (LPT) capacity	Median value of places-km of LPT (bus, tramway, underground, funiculars) per thousand inhabitants	Istat
		ind.66	Rail transport utilisation index	Percentage of workers, schoolchildren and students aged 3 and over who regularly use the train to go to work, kindergarten or school	Istat
		ind.77	Spreading of urban transport	*Urban public transport networks in the provincial capital municipalities for 100 square kilometres of municipal territory*	Istat
		ind.68	Degree of satisfaction with regional rail transport	Average number of people who declare themselves satisfied with the seven different characteristics of the surveyed service (frequency of journeys, punctuality, possibility of finding a seat, cleaning of cars, convenience of the timetables, cost of ticket, information on the service) on the total number of users of the service	Istat
		ind.69	Cycle path density	Median value of the density of km of cycle paths over 100 km.^2^ of the province’s surface	Istat-UrBES
Gender balance (D3.4)		ind.70	Work-life balance for women in the tourism industry	Employment rate of women in the tourism sector (aged 25–49) with pre-school children to women (aged 25–49) without children	Istat
		ind.71	Temporary employment rate for women in the tourism sector	Ratio of no. of female workers (dependent, self-employed, temporary) out of the total employees in the tourism sector (average annual values)	Istat
		ind.72	Female unemployment rate	Total women jobseekers in the female workforce (> 15 years old)	Istat
		ind.73	Long-term female unemployment rate	Women looking for long-term work (> 12 months) out of total women seeking work	Istat
		ind.74	NEET female rate	Percentage of young women (aged 20–34) neither in employment nor in education and training	Istat
		ind.75	Political participation of women	Percentage of female regional councillors elected	Istat-Asvis

The homonymous sub-indexes summarise each of the three dimensions of tourism sustainability. They are adequately organised in pillars, and two (economic and environment) out of three cases, sub-pillars. The economic sub-index captures all the economic output or externalities of tourism activities that contribute to the region development, employment growth, sustainability of tourism enterprises, and international appealing. The environment sub-index identifies tangible and intangible assets related to tourism that usually have an adversarial relationship with the environment. The social sub-index entails aspects of tourism—i.e. human rights (health and security), gender equity, political participation—that affect social communities' sustainability. Each dimension can be considered individually (sub-index) or in combination with the rest of the system (composite indicator), allowing the simultaneous evaluation of all dimensions by linking the regional economy to tourism and its impact on the natural and social environment.

We collected an initial list of 310 elementary indicators. The primary selection criteria (Tanguay et al., 2013) allowed filtering the initial set of indicators (104, 119 and 87 for the economic, environmental and social dimensions, respectively) to obtain a more concise list of 120 indicators based on four principles: (1) relevance of the elementary indicators concerning the three dimensions of sustainable tourism; (2) their frequency of use in the academic research (see Table 1); (3) coverage of the UNWTO's central issues of sustainable development in tourism; (4) their replicability over time. Subsequently, three secondary criteria (Rajaonson & Tanguay, 2012) allowed filtering of the list of 120 indicators to a set of 75 core indicators (34, 21 and 20, respectively): (1) uneven data availability at the regional level; (2) consistency of indicators with the regional policy; (3) relevance of indicators in capturing the territorial heterogeneity among Italian regions.

In addition to tourism-specific indicators, the SusTour-Index also includes a few more general indicators unrelated to tourism but can affect the sustainable development of destinations and the quality of the tourism experience. Torres-Delgado and Lόpez Palomeque (2018) suggested that these indicators—although not directly related to tourism—were kept as they capture relevant aspects of sustainability that are likely to impact or be modified by the tourism activity (see Tanguay et al., 2013). Their relevance to appropriately characterise the three dimensions considered for tourism management justifies the inclusion in the system (Lozano-Oyola et al., 2019b).

The conceptual framework of the SusTour-Index was validated in the Italian regions. The candidate elementary indicators for all 21 regions were obtained from the main national (Istat, ISPRA, Bank of Italy) and international (Eurostat, UNESCO, Foundation for Environmental Education) institutional sources.

The hierarchical structure of the SusTour-Index required elementary indicators to be first synthesised into the pillars (I level) and then the pillars into their sub-index (II level). It can imply multiplicative effects on subjective judgments that involve each step of the construction process (Becker et al., 2017; Mayer, 2008; Singh et al., 2009). This is why we did not choose any model of the SusTour-Index a priori. We used the multi-modelling approach to assess the robustness of the composite indicator by performing a set of 23 models of the SusTour-Index, each obtained as an appropriate mixture of weighting and aggregation schemes consistent with the underlying theoretical framework.

### Data Treatment, Multivariate Analysis and Normalisation

The third step concerns the strategies for handling missing data and outliers. In particular, outliers were detected by comparing the absolute values of skewness and kurtosis of each elementary indicator, respectively, with the critical thresholds of 2 and 3.5 (OECD, 2008).

The fourth step assesses the statistically-determined structure of the data set to the theoretical framework. Cross-correlation analyses between elementary indicators within each pillar and between sub-pillars allowed checking whether the nested structure of the SusTour-Index was well defined (Booysen, 2002; Saisana & Philippas, 2012). Moreover, Principal Component Analysis (PCA) verified the suitability of the data structure to the theoretical framework, and Cronbach's alpha assessed the pillars' internal consistency. An acceptable alpha should range between 0.70 and 0.90, as higher values may suggest that some indicators in the construct are redundant (Tavakol & Dennick, 2011). However, some scholars suggested that alpha values lower than 0.70 could be accepted (Bonett & Wright, 2015; Spiliotopoulou, 2009).

The fifth step makes the indicators with different units of measurements and/or orders of magnitude dimensionless and, therefore, comparable with each other. Elementary indicators were standardised using the adjusted *z*-scores, which involve the use of the traditional standardisation formula for each indicator $\left( {I_{q} } \right)$ adjusted so that the average is 100 and the standard deviation 10:

1 $$z_{qr} = \frac{{x_{qr} - \overline{x}_{q} }}{{\sigma_{q} }} \cdot 10 \cdot d + 100$$

where $\overline{x}_{q}$ and $\sigma_{q}$ are, respectively, the average and the standard deviation of $I_{q}$; *d* stands for the directional adjustment which allows indicators to be corrected when their polarity is discordant with the direction of the latent pillar or sub-pillar they contribute to measuring; *d* is equal to 1 if higher values of the indicator denote better conditions ('the bigger, the better') and -1 otherwise ('the bigger, the worst'). The adjusted *z*-score allows indicators to be converted to a common scale, preserving the relative distances. It shifts the measurement scale on the positive axis, avoids the negative scale problem for geometric aggregation, and is compatible with the aggregation methods performed in the later steps, i.e. the Mazziotta-Pareto aggregation function (Mazziotta et al., 2010).

### Weighting and Aggregation

While the weighting criteria allow assigning weights to each indicator and/or pillar/sub-pillar, the aggregation methods enable elementary indicators to be progressively summarised according to the structure of the composite indicator. When used in a benchmarking framework, weighting and aggregation schemes require caution due to the effects on the composite indicator (Saisana et al., 2011). This is particularly true for the SusTour-Index due to its hierarchical structure, which requires a two-level aggregation procedure (indicators and pillars) using weights at each level.

Weighting methods' choice was strongly dependent on their compatibility with the aggregation methods (OECD, 2008).^2^ In particular, we tested:

1. Equal weighting (EW), which does not mean any weight. A weighting system is implicitly introduced by the informative value of each indicator. EW is a homogeneous weight system and not an absence of weights (OECD, 2008).
2. A data-driven weighting procedure based on principal component analysis (PCA). It allows weights to be obtained endogenously based on data correlation. The rationale of PCA is to group individual indicators that share a large amount of common variance (Mikulić et al., 2015). Indeed, PCA synthesises the original *m* standardised variables in a smaller number $k$ ($< m$) of uncorrelated variables (principal components, PCs), which are the new indicators that explain most of the observed variance. If $k > 1$, the weights of the $j$-th component is equal to the related eigenvalue $\lambda_{j}$ rescaled for the total explained variance $\mathop \sum \limits_{j \le k}^{ } \lambda_{j}$ (normalised variability explained by the *j*-th component); while if $k = 1$, the weight is equal to the factor loadings, scaled to unity sum. The PCA weighting method considers the correlation among the indicators, providing a weighting system for the components to their relevance (Castellano & Rocca, 2019; Munda, 2016).

As regards aggregation, we used: (1) linear aggregation; (2) geometric aggregation; (3) Mazziotta-Pareto (MP) method; (4) Wroclaw taxonomic approach; (5) Borda's rule. The first four allow the metric structure of the indicators to be preserved (cardinal approaches) since the differences between the regions can be expressed in terms of index scores. Borda's rule replaces the index scores with the rankings (ordinal approach), losing the magnitude of differences between regions.

The linear aggregation consists of computing the arithmetic mean of elementary indicators (or pillars) for each pillar (or each sub-index). However, full compensability could affect it, i.e. poor performance in some indicators can be compensated by sufficiently high values of other indicators. Geometric aggregation ensures that there is no possibility of full compensability of low results in one indicator with high results in other indicators.

The MP method is a non-linear aggregation approach based on the hypothesis that elementary indicators are not substitutable (i.e. they do not compensate each other). This involves the introduction of a 'penalty' for entities not showing balanced values of elementary indicators (Mazziotta & Pareto, 2016; Mazziotta et al., 2010). The MP method aggregates standardised indicators by using the arithmetic mean adjusted by a penalty coefficient, which captures the variability of each unit across the set of indicators (i.e. horizontal variability).

Wroclaw's taxonomic approach consists in ranking units in relation to their Euclidean distance from the ideal unit, which is the unit with the best value for that indicator:

2 $$D_{ij} = \sqrt {\mathop \sum \limits_{q = 1}^{Q} \left( {x_{iq} - x_{jq} } \right)^{2} }$$

where $x_{iq}$ is the value of unit *i* for the indicator *q* and $x_{jq}$ is the value of the ideal unit for the same indicator *q*.

Borda's method allows overcoming the problem of 'plurality rule' (i.e. the winner is the unit more often ranked in the first position), declaring the unit with the highest total score as the winner (OECD, 2008). Borda's method aggregates *n* units sorting them into binary relations for pairwise comparisons and assigns a score to each unit in relation to the position assumed in the ranking for each pillar:

3 $$B_{i} = \mathop \sum \limits_{k = 1}^{n} \left( {n - k} \right) \cdot S_{k}$$

where $S_{k}$ shows the number of times that the unit *i* is at the $k_{th}$ position, $\left( {n - k} \right)$ is the corresponding score, while the final rank is based on the total score $\left( {B_{i} } \right)$ received by each unit.

## Results and Discussion

### The Conceptual Structure of the SusTour-Index

The SusTour-Index is composed of 75 elementary indicators at the regional level referring to the year 2017 and organised into pillars and sub-pillars within the economic (D1), environmental (D2) and social (D3) sub-index. The economic dimension consists of 34 elementary indicators, structured into five pillars (Table 2): Economic value (7 elementary indicators); Sustainability of tourism enterprises (5); International appeal of tourism, which in turn is organised in the two sub-pillars of International tourism demand (4) and International tourism satisfaction (4); Tourism demand (6); Cultural heritage organisations (8).

The environmental dimension shows 21 elementary indicators (Table 3). It is structured into four pillars: Energy and water consumption (5 indicators); Sustainable energy management (4); Waste management (6); Environmental value with the two sub-pillars of quality of the environment (3) and Promotion of natural heritage (3).

The social dimension is composed of 20 elementary indicators (Table 4), structured into four pillars: Security (6 indicators); Health (3); Mobility (5); Gender balance (6).

### Measuring the Dimensionality of the SusTour-Index

The institutional nature of the statistical sources ensured high data quality with only 21 missing values (1.3% of total data), mostly concerning Aosta Valley and the autonomous provinces of Trento and Bolzano. The imputation strategy depended on the type of missing data: the regional value was split for count data, and the regional median value was used for score variables. One outlier was detected for eight indicators and two outliers for one indicator, which were winsorised (suggested when outliers are less than 5%), except for one case that was treated through the Box-Cox transformation.

The set of elementary indicators that best converge with the theoretical framework was identified through an iterative procedure, avoiding negative correlations, low correlations ($\rho < 0.33$) and very high correlations ($\rho > 0.92$) (OECD, 2008). This thumb rule required a case-by-case analysis because even if the correlation was outside the range, some indicators were kept when considered crucial in the conceptual structure. The detailed analysis of the correlation structure within and between pillars confirms the higher correlation of each indicator to its pillar than any other, suggesting that the allocation of the elementary indicators to a specific pillar, inside each dimension of the SusTour-Index, is consistent both from a theoretical and statistical perspective (Saisana et al., 2020). As shown in Table 5 (third column), all the Cronbach's alpha values fall between acceptability ranges, except for the 'Environmental value'. PCA confirmed the suitability of the data's underlying structure to the theoretical framework and, therefore, the unidimensional latent structure for each pillar (Table 5, fourth and fifth columns). All eigenvalues are higher than 1, and one component captures more than 70% of the total variance within each pillar, except a few cases (i.e. 'Sustainability of tourism enterprises', 'International appeal', 'Environmental value') for which two components are required. Multivariate analysis was not performed on the pillars of 'Sustainable energy and water management' and 'Health' because of the low number of elementary indicators (fewer than 5 indicators are also critical for computing Cronbach's alpha).

**Table 5:** Statistical dimensionality of the SusTour-index structure

	Pillar	Cronbach’s alpha	Eigen value ($\lambda_{i}$)	Cumulative variance
D1	*Economic sub-index*			
D1.1	Economic value	0.9378	5.086	0.8059
D1.2	Sustainability of tourism enterprise	0.8229	5.4663	0.6192
D1.3	International appeal	0.8541	4.0408	0.5879
			2.0387	0.8845
D1.4	Tourism demand	0.7456	2.9055	0.7148
D1.5	Cultural heritage	0.8812	4.3637	0.7353
D2	*Environmental sub-index*			
D2.1	Energy and water consumption	0.8648	3.1617	0.9605
D2.2	Sustainable energy and water management	–	–	–
D2.3	Waste management	0.7293	2.3033	0.6803
D2.4	Environmental value	0.5864	1.8312	0.446
			1.6619	0.854
D3	*Social sub-index*			
D3.1	Security	0.916	4.1757	0.7635
D3.2	Health	–	–	–
D3.3	Mobility	0.7695	2.2493	0.7922
D3.4	Gender balance	0.8627	3.3907	0.8743

### Weighting and Aggregating: The Multi-modelling Approach

Once all the indicators were made dimensionless, the two-level aggregation procedure allowed elementary indicators to be summarised into the pillars (I level) and the pillars into their sub-index (II level). By adequately combining the weighting and aggregation methods (sub-Sect. 3.3) in both levels, 23 different models of the SusTour-Index were estimated for the Italian regions.

Table 6 details the weighting system and the aggregation procedure used for each model of the SusTour-Index and for both its levels in compliance with the OECD's compatibility criteria (2008). Two sets of models of the SusTour-Index can be distinguished. The first set of 14 models (M1–M14) (Table 6, left panel) was estimated using EW on both levels of aggregation and combining three aggregation methods on the first level (linear, geometric, MP) and five aggregation methods on the second level (linear, geometric, MP, Wroclaw, Borda). On the other side, the introduction of the PCA weighting method on at least one aggregation level, combined with different aggregation techniques, allowed us to estimate another set of 9 models (M15-M23) (Table 6, right panel) of the same composite indicator. In particular, the models of the SusTour-Index from M15 to M17 have EW on the first level and PCA weighting on the second one; the models from M18 to M22 use PCA weighting on the first level and EW on the second one. The last model M23 uses PCA weighting on both levels of aggregation.

**Table 6:** Multi-modelling approach

First set of 14 models (M1–M14)					Second set of 9 models (M15–M23)				
Model	I level (Indicators)		II level (Pillars)		Model	I level (Indicators)		II level (Pillars)	
	Weight	Aggregation of indicators	Weight	Aggregation of pillars		Weight	Aggregation of indicators	Weight	Aggregation of pillars
M1	EW	Linear	EW	Linear	M15	EW	Linear	PCA	Linear
M2	EW	Linear	EW	Geometric	M16	EW	Geometric	PCA	Linear
M3	EW	Linear	EW	MP	M17	EW	MP	PCA	Linear
M4	EW	Linear	EW	Wroclaw	M18	PCA	Linear	EW	Linear
M5	EW	Linear	EW	Borda	M19	PCA	Linear	EW	Geometric
M6	EW	Geometric	EW	Linear	M20	PCA	Linear	EW	MP
M7	EW	Geometric	EW	Geometric	M21	PCA	Linear	EW	Wroclaw
M8	EW	Geometric	EW	MP	M22	PCA	Linear	EW	Borda
M9	EW	Geometric	EW	Wroclaw	M23	PCA	Linear	PCA	Linear
M10	EW	Geometric	EW	Borda					
M11	EW	MP	EW	Linear					
M12	EW	MP	EW	Geometric					
M13	EW	MP	EW	Wroclaw					
M14	EW	MP	EW	Borda					

### Assessing Reliability and Validity

Once the 23 models were performed, sensitivity analysis allowed us to evaluate the robustness of the SusTour-Index. Sensitivity is closely related to uncertainty analysis, which allows quantifying the impact of weighting and aggregation choices on the variation in the regions' rankings (scores), contributing to SusTour-Index well-structuring and improving the consistency of the results.

Descriptive statistics on rankings of Italian regions obtained from the 23 different models of the SusTour-Index represent a first way to evaluate the outcomes' stability. “Appendix 1” shows both synthesis measures and variability of the positions occupied by each region as the models (weighting and aggregation methods) change, allowing an evaluation of the criticalities in the construction of the SusTour-Index in terms of output instability. Tables 7, 8 and 9 also show the 95% confidence intervals of the median rank for each region, using bootstrap procedures (2000 samples) (Efron & Tibshirani, 1998) to measure the volatility of the rankings due to a change to the underlying methodology (Saisana et al., 2011).

Statistics show lower sensitivity for the first set of models in which the EW system was used than for the second set of models estimated using the PCA weighting method. The analysis of the central tendency (mean; median) and variability (standard deviation, SD; coefficient of variation, CV; median absolute deviation, MAD) of regional rankings estimated by the two sets of models of the SusTour-Index shows a strong agreement in the rankings among EW models (M1–M14) for each of the three sub-indexes. In particular, as regards the environmental and social sub-index, no region shows an SD of rankings higher than 3 if EW models were used, while all regions (except one for the social dimension) would exceed this SD value in the case of models with PCA weights (Tables 8, 9). Moving on to the economic sub-index, the SD higher than 3 is confirmed for more than 70% of regions if models with PCA weights were used, against 15% in the case of EW models (Table 7). Therefore, a higher variability is detected for the second set of models (M15-M23) for which the regional rankings are more sensitive to the chosen weighting and aggregation methods. The lower ranking stability resulting from models using the PCA weighting procedure is also shown by MAD. These results are also confirmed by the estimated confidence intervals, which are consistently lower for the first set of models (EW) for each dimension of tourism sustainability. As expected, lower volatility is observed for the top and bottom regions of the ranking (i.e. the best and worst performers are essentially the same regions across the different models of the SusTour-Index), while greater differences are found for the middle ranking regions.

Spearman's rank correlation helps measure the proximity of rankings obtained from the different models (“Appendix 2”). The Spearman coefficients, separately computed for the economic, environmental and social sub-index (Tables 10, 11, 12), are consistently positive for the EW models (M1–M14). Moreover, the methods of aggregation between the pillars (level II) based on linear and geometric aggregations are the most correlated with the remaining methods. With an average Spearman's rank coefficient higher than 0.90 within each sub-index, the rankings from the EW models are highly correlated to each other. In particular, the rankings from models combining EW methods with linear (M1–M5) or MP (M11–M14) aggregation on the first level are very similar. In fact, although a few differences are highlighted among middle-ranking regions, the best and worst performers are substantially the same regions across all these methods.

On the other side, rankings from the second set of models in which data-driven weights based on PCA were used (M15–M23) are much less correlated with each other, with Spearman coefficients even negative (Tables 10, 11, 12), and less correlated with those of the first set. More particularly regardig the economic sub-index, the most problematic models (M16, M18 and M19) all belong to the second set, which used PCA weights in one of the two levels of aggregation and EW with linear or geometric aggregation in the other level. Similarly, moving on to the environmental sub-index, problems arise for M17 and M23 whose rankings are negatively correlated with those from each other model. For the social sub-index, with few exceptions, most models (M15, M17, M18, M19, M23) show negative correlations with the other ones, especially with the EW models. The rankings from the M20-M22 are positively correlated with each other and, more moderately, with the EW models.

In a nutshell, models using PCA weights in at least one of the two hierarchical levels are less correlated with each other, regardless of the aggregation function adopted, letting us prefer EW models. Therefore, the models of the SusTour-Index with equal weights in both aggregation levels appear to be more suitable than those weighting the indicators through the PCA procedure.

### Assessing Sustainable Tourism in the Italian Regions

One of the most relevant results of this research is that the proposed structure of the SusTour-Index contributed to making the rankings from different models robust, and rankings attained by the set of EW models are quite similar to each other. *Ceteris paribus*, this would allow the selection of those models of the SusTour-Index which are most correlated with all the others.

The M2 model was used to assess and discuss the Italian regions' relative performance in tourism sustainability. M2 model performs the linear aggregation in the first level and geometric aggregation in the second level. However, we would like to reiterate that one EW model of the SusTour-Index is as good as another given the high correlation between the rankings. Given the hierarchical structure of the SusTour-Index, a third step was required to aggregate through geometric mean the three sub-indexes—economic, environmental and social—into the overall Sus-Tour Index to provide insights into the broader tourism sustainability.

**Fig. 2:**
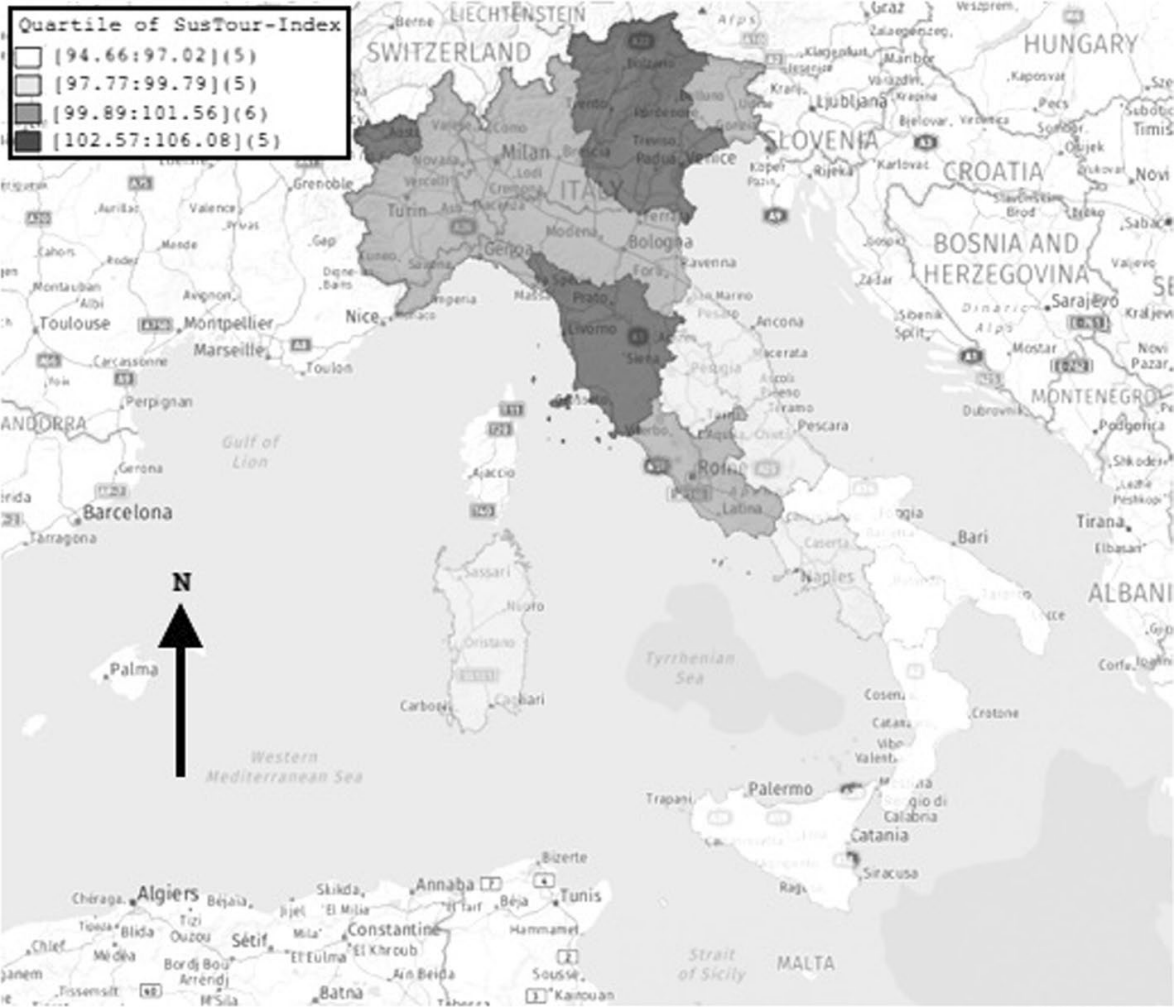
Map of Italian regions by quartile of SusTour-Index score

Considering the overall sustainable tourism development (M2 model), four clusters of regions can be identified (Fig. 2):

1. *Very low* scores (I quartile): Apulia, Basilicata, Molise, Calabria, and Sicily;
2. *Low* scores (II quartile): Abruzzo, Marche, Campania, Umbria, and Sardinia;
3. *High* scores (III quartile): Emilia Romagna, Friuli-Venezia Giulia, Latium, Liguria, Lombardy, and Piedmont;
4. *Very high* scores (IV quartile): Bolzano, Trento, Aosta Valley, Veneto, and Tuscany.

Tourism sustainability moves from the highest levels in the northern and central regions towards the lowest levels in the southern regions. The historical pattern of the territorial divide between northern and southern Italy re-emerges as indicated by the overall SusTour-Index, the three sub-indexes and the elementary indicators.

**Fig. 3:**
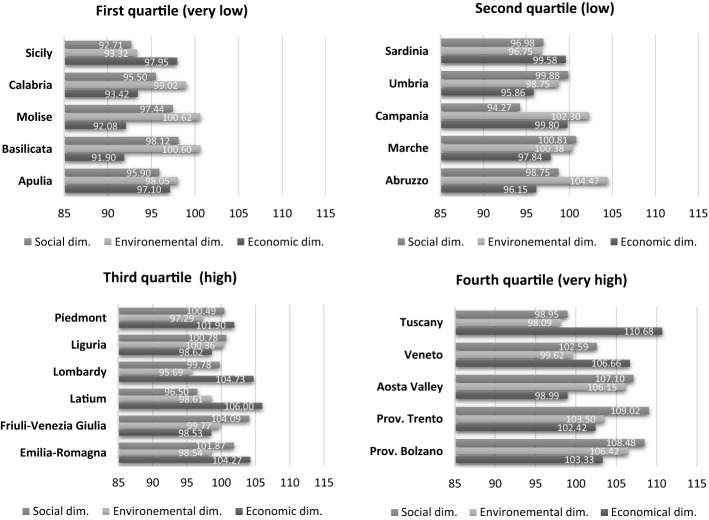
Italian regions by quartile of the overall SusTour-Index scores

Looking within each cluster of regions by quartile of the overall SusTour-Index score, it is worth noting the relatively homogeneous rankings for the three dimensions of tourism sustainability (Fig. 3).

The first two clusters present low tourism sustainability performances on each sustainability dimension. Molise and Basilicata keep better rankings in the environmental dimension (first quartile); Abruzzo and Campania are very close to the regions with a higher SusTour-Index (second quartile). Although high economic and social sustainability levels characterise the regions with a relatively high overall SusTour-Index (third quartile), they lack environmental sustainability (with the lowest level in Lombardy). The regions with the highest SusTour-Index (fourth quartile) perform very well in all dimensions, with Trento, Bolzano and Aosta Valley at the top of environmental and social sustainability.

The three sub-indexes and their elementary indicators evaluate possible drivers and reasons for the top regional positioning in sustainable tourism development.

*Economic sustainability* reflects the Italian economic scenario, characterised by the historic north/centre-south territorial divide (Castellano et al., 2016). The best regions (Tuscany, Veneto, Latium, and Emilia-Romagna) present a long-term economic development based on consolidated tourism activities and cultural heritage, which enhance the international tourism demand.

*Sustainability of tourism enterprise* (D1.2) plays a relevant role in Tuscany, Veneto, Latium, Emilia-Romagna and Lombardy, as specific elementary indicators indicate: tourism companies (ind.08), accommodation establishments (ind.09), ISO 14001 certification (ind.10). In Tuscany, Emilia-Romagna and Lombardy, additional value has been generated by EMAS registration (ind.11); Ecolabel license (ind.12) presents important values for Tuscany and Emilia-Romagna.

All regions show higher *tourism arrivals* (ind.21) and *tourism presence in accommodation establishments* (ind.22). They attract *international tourism demand* (D1.3.1), excluding Emilia-Romagna, as some indicators suggest: tourist stay of foreigners (ind.13), quota of foreign tourism on domestic tourism (ind.14) (excluding Lombardy), foreign tourism expenditure (ind.15), and foreign tourists revisiting a place (ind.16). *Cultural heritage organisations* (D1.5) represent other key elements of economic sustainability of the best Italian regions: heritage museum (ind.30), degree of promotion of the cultural offer of the state institutes (ind.32) except for Emilia Romagna, cultural events (ind.33), expenditure for cultural events (ind.34). Museum visitors (ind.25) and cultural heritage visitors (ind.26) are relevant drivers for Tuscany and Latium.

The southern regions have not yet managed to exploit the full potential of tourism for the sustainable development of the local economy. On the contrary, Campania, Sicily and Sardinia show a fair performance for tourism's economic sustainability, as characterised by important levels of diverse indicators.

*Environmental sustainability* appears patchy with some best performers: Abruzzo, Aosta Valley, Bolzano, Campania, Trento, and Molise. Their relatively low environmental impact has been driven by several key aspects, which show diversity in northern and southern regional behaviours. Reducing energy and water consumption (D2.1: ind35- ind38), sustainable energy and water management (D2.2: ind40- ind43) and waste management (D2.3: mainly ind48) represent the main investments of the northern regions, such as Aosta Valley, Bolzano and Trento. Molise adopts a similar approach in reducing energy and water consumption (D2.1: ind35- ind38).

The southern regions, Campania and Molise, reach their advantageous position for environmental sustainability by investing in the territory's environmental value (D2.4). It considers aspects related to the quality and promotion of the environment, such as the certification of the Blue Flag beach (only Campania, ind.50), favourable conservation of the habitat (ind.51), quality of bathing coasts (ind.52), sites of Community Importance (ind.53), Special Protection Area (ind.54) and Rete Natura (ind.55). Aosta Valley, Bolzano and Trento also excel in the sites of Community Importance (ind.53) and in the Special Protection Area (ind.54); Aosta Valley even in Rete Natura (ind.55).

The *social sustainability of tourism* has been concentrated in the north-east of Italy (Trento, Bolzano, Veneto, and Friuli-Venezia Giulia) and the Aosta Valley. These regions represent the best in almost all elementary indicators, presenting high family health care expenditure (ind.64) and cycle path density (ind.69). Furthermore, Trento, Bolzano and Aosta Valley have invested in hospitals (ind.63), while Bolzano, Friuli Venezia Giulia and Aosta Valley are spreading urban transport (ind.67). Gender balance (D3.4: ind.72–74) represents a key element of social sustainability in Trento and Bolzano.

## Conclusion

This paper proposed a multi-modelling approach for building and validating a new composite indicator of sustainable tourism, the SusTour-Index, offering theoretical and methodological contributions and opening rooms for future research. Besides, it provides practical advancements in measuring tourism sustainability, supporting policymakers, institutions and practitioners in planning and managing tourism development.

From a theoretical perspective, the SusTour-Index implements a comprehensive framework to summarise the multidimensionality of tourism sustainability with a broad set of elementary indicators, effectively structured in pillars and sub-pillars within each sustainability dimension (economic, environmental, social). The rationale of the SusTour-Index required relevant indicators that captured the territorial heterogeneity across regions, avoiding misleading indicators for policy implications.

The multi-modelling approach validated the hierarchical structure of the SusTour-Index in all 21 Italian regions on a range of 23 alternative models, which allowed evaluating the impact of the different methodological choices in dealing with the elementary indicators inside each sustainability dimension. A notable strength of the SusTour-Index is represented by the overcoming of the close dependence on specific local destinations. The use of objective indicators from institutional sources made it possible to capture the economic, environmental, and social diversity of sustainable tourism among the Italian regions. The conceptual framework of the SusTour-Index can support policymakers as it also ensures comparability between regions and replicability over time.

From a methodological perspective, evaluating how a choice in dealing with elementary indicators affects the outcomes (scores and rankings) of the SusTour-Index provided new insights into this issue. The use of equal weights for sustainability indicators appeared to be a better strategy than potentially altering some indicators' importance through data-driven weighting approaches. The multi-modelling analysis also demonstrated the lower sensitivity of EW models to aggregation functions compared to models in which PCA weights were used. The aggregation functions used to build the SusTour-Index could be considered exchangeable when using the EW approach to facilitate their interpretation (Singh et al., 2009).

Some past research (see Mikulić et al., 2015) proved the opportunity of using equal weights for sustainability indicators in certain circumstances but relying exclusively on an illustrative comparison of indicator weights from available studies. This research made further efforts to verify the low sensitivity of EW systems by performing simulations on the same set of elementary indicators. The proposed framework of the SusTour-Index contributed to making the rankings of the different EW models robust; that is, the rankings (scores) obtained from the set of EW models are quite similar to each other. Given their interchangeability, therefore, it is possible to choose the model of the SusTour-Index that is most correlated with all the others. From a practical perspective, the SusTour-Index contributes to monitoring, planning, and managing the critical aspects of sustainable development, helping institutions and policymakers address effective and coherent policies towards the SDGs. The results on the Italian regions underline the value of the SusTour-Index, which can support practical assessments of tourism impacts and design evolutionary scenarios for sustainable destination development. The structure of the SusTour-Index can define specific dashboards for decision-makers and institutions to monitor the impact of economic tourism activities and their implications on the environment and society. An in-depth analysis can introduce strategic interventions, investments and policies that help redress the economic, social and environmental imbalances towards SDSs. Comparing performances across all dimensions identifies the best practices in pursuing tourism sustainability and possible actions to manage critical events and behaviours and invest in destinations' key pillars of sustainable development.

The research proposes to differentiate interventions and public investments by considering the value of overall sustainable tourism development (M2 model) in each Italian region combined with specific elementary indicators' role (and value) in the regional strategic plan. Overcoming generalised interventions for regional sustainable development, the SusTour-Index can direct and redirect specific strategies to optimise the allocation of public funds. In light of the overall value of the sustainable indicators and specific assessment of the elementary indicators, Italian institutions and policymakers could more efficiently implement NGEU (NextGenerationEU) investments and PNRR (National Recovery and Resilience Plan) strategies and actions. They can redirect funds and investments, taking into account the values of the indicators and the sustainable development goals to be achieved in each region.

Future research may consider the emerging role of technologies in addressing and managing sustainable tourism. Technology-driven innovation can enhance sustainability by addressing stakeholder (e.g. tourists, residents, workers, entrepreneurs) behaviours and managing tourism imbalances (e.g. overcrowding, gentrification, etc.). Smart technology tools can support sustainable decision-making, providing solutions to implement adaptive actions, such as redirecting and dispersing tourist flows from icon sites and attractions. For example, an app can offer users alternative routes and locations to visit a city when the number of tourists exceeds the carrying capacity of a specific area. Furthermore, digital technologies (e.g. social media campaigns) can create preferences and shape sustainable behaviours in destinations.

By advancing tourism planning and management, the SusTour-Index supports effective governance (institutions, destination management organisations, policymakers) in achieving sustainable tourism development by: monitoring the impacts and imbalances of tourism development, defining stakeholder rules, policy evaluations and monitoring practices; creating contingency plans for peak periods, emergencies and crisis; defining managerial tools to support destination decisions; introducing a valuable information system for destination stakeholders, including institutions, policymakers, tourism enterprises, community and other key players.

Interpreting the SusTour-Index as a tool for policy learning and policy change in societal behaviours can open new rooms for advances in participatory sustainable development that have received marginal attention (Rasoolimanesh et al., 2020). Stakeholders' engagement in sustainable development goals requires calibrating political-institutional strategies and actions with an inclusive and shared vision of sustainable development (Omrani et al., 2019; Pasquinelli & Trunfio, 2020).

While providing interesting food for future research, the present work does not come without its limitations. As the regional level is considered, weighting approaches adopted do not consider performance levels of sustainability indicators for each destination separately. Still, they take indicator performance levels for multiple destinations to obtain the weights. Therefore, further research is needed to establish guidelines for the weighting and aggregation of tourism sustainability indicators at different territorial levels (e.g. country, municipality, destination) while preserving their comparability and replicability. Moreover, this paper also calls for empirical validation in other countries, which can enhance the value of the conceptual framework of the SusTour-Index by enabling shared theoretical advances and practical tools for policymakers.

Last but not least, in the post-COVID-19 time, the conceptual framework of the SusTour-Index can offer new challenges for scholars called to shift from over-tourism to under-tourism. An efficient transition and crisis management will require a solid set of indicators to assess and monitor the tourism impact on regions and destinations. Future research could develop the conceptual framework of the SusTour-Index by including an additional dimension that measures the health and sanitary security protocols, opening new interdisciplinary research streams.

## Appendix 1

See Tables 7, 8 and 9.

**Table 7:** Economic sub-index: summary statistics

Regions	Model with EW						Data-driven weighting models					
	Mean	Median	SD	CV	MAD	95% CI*	Mean	Median	SD	CV	MAD	95% CI*
Piedmont	10.43	9	3.44	0.33	1.48	[7.5; 10.5]	9.89	8	6.30	0.64	1.48	[5; 11]
Aosta Valley	14.71	14.5	2.92	0.20	3.71	[13; 16]	8.78	8	5.69	0.65	4.45	[6; 10]
Liguria	13.14	12	3.18	0.24	2.22	[11; 13]	10.89	11	4.12	0.38	2.97	[9; 13]
Lombardy	6.14	5	3.84	0.63	0.74	[3; 7]	7.89	4	7.50	0.95	4.45	[1; 7]
Bolzano	6.36	6	0.74	0.12	0.00	[6; 6]	4.89	5	4.51	0.92	4.45	[3; 7]
Trento	7.71	7	1.33	0.17	0.74	[6; 8]	4.67	3	3.77	0.81	2.97	[1; 5]
Veneto	2.43	2	0.65	0.27	0.00	[2; 2]	7.00	5	5.42	0.77	4.45	[3; 7]
Friuli-Venezia Giulia	12.93	13	1.54	0.12	0.74	[12; 14]	13.00	13	2.91	0.22	4.45	[12; 14]
Emilia-Romagna	4.86	4	2.38	0.49	0.00	[3; 5]	8.22	5	7.41	0.90	5.93	[2; 8]
Tuscany	1.00	1	0.00	0.00	0.00	[1; 1]	4.44	1	6.27	1.41	0.00	[1; 3]
Umbria	16.71	18	2.67	0.16	0.00	[17; 19]	13.78	16	4.78	0.35	2.97	[14; 18]
Marche	13.21	13.5	1.19	0.09	2.22	[13; 14]	12.67	14	2.26	0.18	1.48	[13; 15]
Latium	3.29	3	2.27	0.69	0.00	[2; 4]	6.11	3	6.19	1.01	2.97	[1; 5]
Abruzzo	16.14	17	1.35	0.08	0.74	[16; 18]	15.56	17	2.79	0.18	1.48	[16; 18]
Molise	19.86	20	1.46	0.07	0.00	[19; 21]	17.33	20	5.66	0.33	1.48	[18; 22]
Campania	8.07	8.5	2.40	0.30	2.22	[7.5; 10.5]	11.44	12	2.36	0.21	2.97	[11; 13]
Apulia	14.00	15	2.54	0.18	1.48	[14; 16]	15.11	16	2.60	0.17	1.48	[15; 17]
Basilicata	20.71	21	0.47	0.02	0.00	[21; 21]	16.78	20	6.37	0.38	1.48	[17; 23]
Calabria	18.71	19	0.61	0.03	0.00	[19; 19]	15.00	19	5.58	0.37	0.00	[17; 21]
Sicily	12.64	14	2.87	0.23	2.22	[13; 15]	12.22	12	1.75	0.14	1.48	[11; 13]
Sardinia	7.71	8	2.46	0.32	2.97	[7; 9]	9.11	10	4.95	0.54	5.93	[8; 12]

*95% confidence interval of the median rank

**Table 8:** Environment sub-index: summary statistics

Regions	Models with EW						Data-driven weighting models					
	Mean	Median	SD	CV	MAD	95% CI*	Mean	Median	SD	CV	MAD	95% CI*
Piedmont	17.14	18	1.60	0.09	0.14	[17; 19]	14.89	15	3.66	0.25	0.36	[13; 17]
Aosta Valley	2.50	2.5	0.91	0.36	0.54	[2; 3]	6.11	2	7.64	1.25	1.85	[1; 5]
Liguria	10.86	10	2.82	0.26	0.39	[9; 11]	11.44	12	5.78	0.50	0.75	[9.5; 14.5]
Lombardy	19.93	20	0.80	0.04	0.06	[20; 20]	11.33	16	9.13	0.81	1.19	[12; 20]
Bolzano	3.29	3	1.62	0.49	0.73	[2; 4]	7.33	3	8.14	1.11	1.65	[1; 6.5]
Trento	4.36	4	0.81	0.19	0.28	[4; 4]	9.89	10	6.10	0.62	0.91	[7; 13]
Veneto	12.29	12	2.49	0.20	0.30	[11; 13]	8.89	11	7.42	0.83	1.24	[8; 14]
Friuli-Venezia Giulia	12.64	11.5	2.47	0.20	0.29	[10; 13]	7.56	6	6.18	0.82	1.21	[3; 9]
Emilia-Romagna	15.86	16	1.19	0.07	0.11	[15.5; 16.5]	11.56	13	5.46	0.47	0.70	[11; 15]
Tuscany	13.36	13.5	2.50	0.19	0.28	[12; 15]	13.44	15	4.00	0.30	0.44	[13; 17]
Umbria	13.79	13	1.74	0.13	0.19	[12; 14]	12.44	12	3.24	0.26	0.39	[11; 13]
Marche	7.50	8	1.45	0.19	0.29	[7; 9]	10.78	11	2.39	0.22	0.33	[10; 12]
Latium	13.64	14	1.95	0.14	0.21	[13; 15]	13.22	16	5.59	0.42	0.63	[14; 18]
Abruzzo	1.43	1	0.73	0.51	0.76	[1; 1]	4.00	3	3.86	0.96	1.43	[1; 5]
Molise	5.57	6	1.92	0.34	0.51	[5; 7]	7.89	8	5.70	0.72	1.07	[6; 10]
Campania	4.14	5	1.25	0.30	0.45	[4.5; 5.5]	6.78	6	4.24	0.63	0.93	[4; 8]
Apulia	14.43	14.5	2.69	0.19	0.28	[13; 16]	12.78	11	5.33	0.42	0.62	[9; 13]
Basilicata	7.21	7	1.01	0.14	0.21	[7; 7]	8.44	11	5.64	0.67	0.99	[9; 13]
Calabria	10.00	10	1.41	0.14	0.21	[9; 11]	13.33	16	4.59	0.34	0.51	[14; 18]
Sicily	20.79	21	0.41	0.02	0.03	[21; 21]	15.00	21	7.23	0.48	0.71	[18; 24]
Sardinia	19.14	19	0.52	0.03	0.04	[19; 19]	14.11	16	4.95	0.35	0.52	[14; 18]

*95% confidence interval of the median rank

**Table 9:** Social sub-index: summary statistics

Regions	Models with EW						Data-driven weighting models					
	Mean	Median	SD	CV	MAD	95% CI*	Mean	Median	SD	CV	MAD	95% CI*
Piedmont	8.29	8	1.33	0.16	0.24	[7; 9]	12.00	15	6.27	0.52	0.77	[12; 18]
Aosta Valley	2.86	3	0.35	0.12	0.18	[3; 3]	6.78	8	4.39	0.62	0.96	[6; 10]
Liguria	9.71	10	1.79	0.18	0.27	[9; 11]	12.56	13	4.74	0.38	0.56	[11; 15]
Lombardy	11.96	11	2.05	0.17	0.25	[10; 12]	15.22	17	6.16	0.40	0.60	[14; 20]
Bolzano	2.14	2	0.35	0.16	0.24	[2; 2]	11.11	13	8.70	0.78	1.16	[9; 17]
Trento	1.00	1	0.00	0.00	0.00	[1; 1]	10.22	14	7.00	0.69	1.02	[11; 17]
Veneto	5.14	5	0.35	0.07	0.10	[5; 5]	12.89	14	4.56	0.35	0.52	[12; 16]
Friuli-Venezia Giulia	4.00	4	0.00	0.00	0.00	[4; 4]	11.78	11	4.64	0.39	0.58	[9; 13]
Emilia-Romagna	7.57	6	2.58	0.34	0.51	[5; 7]	11.22	12	7.84	0.70	1.04	[9; 15]
Tuscany	13.14	12	2.36	0.18	0.27	[11; 13]	14.22	13	3.12	0.22	0.33	[12; 14]
Umbria	9.21	9	1.15	0.12	0.18	[8.5; 9.5]	13.22	13	2.82	0.21	0.32	[12; 14]
Marche	7.07	7	0.46	0.06	0.10	[7; 7]	13.44	12	3.10	0.23	0.34	[11; 13]
Latium	17.64	17.5	0.89	0.05	0.08	[17; 18]	13.67	12	4.37	0.32	0.47	[10; 14]
Abruzzo	11.21	13	3.10	0.28	0.41	[12; 14]	11.89	15	4.20	0.35	0.52	[13; 17]
Molise	14.36	15	1.76	0.12	0.18	[14; 16]	6.11	5	5.76	0.94	1.40	[2.5; 7.5]
Campania	19.36	20	1.49	0.08	0.11	[19; 21]	9.56	7	4.74	0.50	0.74	[5; 9]
Apulia	17.07	18	1.83	0.11	0.16	[17; 19]	12.56	11	6.06	0.48	0.72	[8; 14]
Basilicata	12.86	13.5	2.17	0.17	0.25	[13; 14]	4.00	2	5.12	1.28	1.90	[1; 5]
Calabria	18.71	19	0.96	0.05	0.08	[19; 19]	5.11	3	5.40	1.06	1.57	[1; 5]
Sicily	20.93	21	0.26	0.01	0.02	[21; 21]	10.22	5	8.40	0.82	1.22	[1; 9]
Sardinia	15.86	16	0.99	0.06	0.09	[16; 16]	9.44	9	3.17	0.34	0.50	[8; 10]

*95% confidence interval of the median rank

## Appendix 2

See Tables 10, 11 and 12.

**Table 10:** Spearman rank coefficients between M1–M23 models: Economic sub-index

	M1	M2	M3	M4	M5	M6	M7	M8	M9	M10	M11	M12	M13	M14	M15	M16	M17	M18	M19	M20	M21	M22	M23
M1	1																						
M2	0.999	1																					
M3	0.979	0.978	1																				
M4	0.978	0.981	0.975	1																			
M5	0.972	0.975	0.980	0.968	1																		
M6	0.866	0.864	0.906	0.868	0.919	1																	
M7	0.801	0.799	0.843	0.814	0.873	0.977	1																
M8	0.487	0.481	0.501	0.440	0.515	0.591	0.629	1															
M9	0.834	0.830	0.870	0.827	0.897	0.984	0.982	0.596	1														
M10	0.888	0.884	0.916	0.884	0.926	0.986	0.955	0.633	0.960	1													
M11	0.999	0.997	0.978	0.979	0.973	0.868	0.806	0.495	0.836	0.890	1												
M12	0.994	0.995	0.988	0.988	0.984	0.877	0.816	0.475	0.843	0.893	0.995	1											
M13	0.981	0.983	0.978	0.999	0.966	0.857	0.797	0.431	0.814	0.876	0.982	0.991	1										
M14	0.971	0.973	0.982	0.971	0.997	0.924	0.876	0.510	0.902	0.927	0.972	0.984	0.968	1									
M15	0.999	1.000	0.978	0.981	0.975	0.864	0.799	0.481	0.830	0.884	0.997	0.995	0.983	0.973	1								
M16	− 0.860	− 0.858	− 0.882	− 0.861	− 0.919	− 0.979	− 0.978	− 0.649	− 0.978	− 0.977	− 0.865	− 0.869	− 0.848	− 0.919	− 0.858	1							
M17	0.995	0.996	0.974	0.981	0.975	0.861	0.806	0.505	0.830	0.885	0.997	0.995	0.983	0.973	0.996	− 0.866	1						
M18	0.271	0.273	0.214	0.265	0.284	0.200	0.316	0.496	0.251	0.209	0.274	0.252	0.243	0.267	0.273	− 0.322	0.300	1					
M19	0.130	0.136	0.064	0.138	0.124	0.018	0.146	0.369	0.052	0.032	0.132	0.108	0.116	0.103	0.136	− 0.129	0.160	0.913	1				
M20	0.900	0.896	0.873	0.887	0.836	0.708	0.648	0.308	0.666	0.730	0.900	0.890	0.894	0.831	0.896	− 0.689	0.896	0.209	0.112	1			
M21	0.762	0.758	0.801	0.773	0.786	0.873	0.824	0.533	0.803	0.892	0.762	0.766	0.765	0.777	0.758	− 0.831	0.752	0.035	− 0.068	0.734	1		
M22	0.889	0.886	0.864	0.865	0.814	0.663	0.577	0.310	0.606	0.701	0.889	0.880	0.879	0.809	0.886	− 0.637	0.886	0.132	0.031	0.983	0.711	1	
M23	0.982	0.978	0.971	0.952	0.957	0.879	0.819	0.558	0.840	0.906	0.978	0.969	0.953	0.953	0.978	− 0.871	0.973	0.325	0.193	0.888	0.798	0.876	1

**Table 11:** Spearman rank coefficients between M1–M23 models: Environment sub-index

	M1	M2	M3	M4	M5	M6	M7	M8	M9	M10	M11	M12	M13	M14	M15	M16	M17	M18	M19	M20	M21	M22	M23
M1	1																						
M2	0.986	1																					
M3	0.942	0.970	1																				
M4	0.934	0.958	0.953	1																			
M5	0.888	0.916	0.882	0.920	1																		
M6	0.975	0.962	0.905	0.900	0.897	1																	
M7	0.973	0.970	0.923	0.910	0.907	0.994	1																
M8	0.879	0.900	0.943	0.888	0.839	0.896	0.914	1															
M9	0.860	0.875	0.861	0.861	0.844	0.916	0.918	0.934	1														
M10	0.870	0.875	0.865	0.839	0.858	0.875	0.901	0.901	0.866	1													
M11	0.986	0.996	0.962	0.958	0.911	0.957	0.965	0.887	0.853	0.863	1												
M12	0.982	0.995	0.964	0.965	0.912	0.952	0.962	0.888	0.856	0.872	0.997	1											
M13	0.931	0.964	0.969	0.994	0.917	0.897	0.914	0.909	0.870	0.861	0.961	0.968	1										
M14	0.875	0.920	0.901	0.931	0.984	0.863	0.884	0.842	0.832	0.866	0.914	0.918	0.936	1									
M15	0.555	0.642	0.696	0.655	0.701	0.513	0.564	0.574	0.487	0.551	0.622	0.627	0.686	0.752	1								
M16	0.629	0.653	0.661	0.700	0.557	0.601	0.577	0.623	0.669	0.446	0.638	0.626	0.690	0.594	0.497	1							
M17	− 0.647	− 0.670	− 0.684	− 0.788	− 0.619	− 0.591	− 0.568	− 0.597	− 0.626	− 0.471	− 0.666	− 0.670	− 0.764	− 0.646	− 0.530	− 0.905	1						
M18	0.875	0.861	0.847	0.860	0.729	0.782	0.784	0.727	0.630	0.731	0.881	0.887	0.858	0.732	0.423	0.494	− 0.626	1					
M19	0.777	0.782	0.790	0.840	0.735	0.694	0.714	0.722	0.600	0.723	0.809	0.825	0.827	0.757	0.434	0.450	− 0.583	0.881	1				
M20	0.538	0.481	0.355	0.380	0.351	0.588	0.560	0.373	0.447	0.377	0.495	0.496	0.338	0.295	− 0.115	0.134	− 0.092	0.401	0.348	1			
M21	0.559	0.485	0.394	0.388	0.434	0.580	0.552	0.402	0.382	0.492	0.498	0.497	0.353	0.363	− 0.037	0.000	− 0.027	0.458	0.447	0.841	1		
M22	0.492	0.435	0.305	0.337	0.319	0.558	0.525	0.340	0.434	0.332	0.450	0.451	0.296	0.254	− 0.179	0.114	− 0.068	0.362	0.296	0.990	0.810	1	
M23	− 0.253	− 0.174	− 0.091	− 0.223	− 0.052	− 0.240	− 0.160	− 0.100	− 0.223	− 0.030	− 0.183	− 0.184	− 0.151	− 0.014	0.462	− 0.378	0.408	− 0.300	− 0.261	− 0.510	− 0.371	− 0.528	1

**Table 12:** Spearman rank coefficients between M1–M23 models: Social sub-index

	M1	M2	M3	M4	M5	M6	M7	M8	M9	M10	M11	M12	M13	M14	M15	M16	M17	M18	M19	M20	M21	M22	M23
M1	1																						
M2	0.999	1																					
M3	0.995	0.997	1																				
M4	0.965	0.970	0.974	1																			
M5	0.965	0.960	0.949	0.898	1																		
M6	0.922	0.925	0.921	0.930	0.838	1																	
M7	0.922	0.925	0.921	0.930	0.838	1.000	1																
M8	0.897	0.901	0.899	0.916	0.818	0.982	0.982	1															
M9	0.919	0.923	0.925	0.945	0.828	0.964	0.964	0.965	1														
M10	0.902	0.903	0.902	0.856	0.848	0.902	0.902	0.873	0.876	1													
M11	0.997	0.999	0.996	0.964	0.960	0.923	0.923	0.895	0.916	0.906	1												
M12	0.996	0.999	0.999	0.973	0.952	0.926	0.926	0.904	0.929	0.907	0.997	1											
M13	0.956	0.965	0.970	0.981	0.876	0.947	0.947	0.925	0.952	0.869	0.961	0.971	1										
M14	0.979	0.975	0.970	0.917	0.988	0.851	0.851	0.825	0.846	0.879	0.974	0.972	0.903	1									
M15	− 0.761	− 0.762	− 0.764	− 0.721	− 0.776	− 0.555	− 0.555	− 0.565	− 0.560	− 0.518	− 0.755	− 0.756	− 0.684	− 0.788	1								
M16	0.671	0.674	0.673	0.645	0.715	0.436	0.436	0.448	0.457	0.392	0.668	0.664	0.583	0.710	− 0.958	1							
M17	− 0.735	− 0.736	− 0.736	− 0.690	− 0.748	− 0.529	− 0.529	− 0.536	− 0.530	− 0.487	− 0.730	− 0.730	− 0.655	− 0.764	0.994	− 0.965	1						
M18	− 0.448	− 0.425	− 0.417	− 0.401	− 0.432	− 0.358	− 0.358	− 0.338	− 0.368	− 0.214	− 0.414	− 0.416	− 0.391	− 0.449	0.513	− 0.432	0.503	1					
M19	− 0.728	− 0.710	− 0.703	− 0.677	− 0.714	− 0.619	− 0.619	− 0.599	− 0.650	− 0.487	− 0.699	− 0.700	− 0.663	− 0.721	0.675	− 0.596	0.653	0.912	1				
M20	0.547	0.537	0.516	0.396	0.591	0.511	0.511	0.451	0.465	0.711	0.555	0.527	0.425	0.577	− 0.150	0.092	− 0.118	− 0.092	− 0.304	1			
M21	0.412	0.405	0.388	0.312	0.402	0.530	0.530	0.478	0.399	0.669	0.422	0.403	0.372	0.406	0.031	− 0.165	0.068	− 0.011	− 0.130	0.834	1		
M22	0.596	0.589	0.570	0.458	0.636	0.556	0.556	0.498	0.510	0.768	0.605	0.579	0.481	0.625	− 0.209	0.144	− 0.172	− 0.073	− 0.301	0.989	0.828	1	
M23	− 0.647	− 0.656	− 0.661	− 0.613	− 0.669	− 0.423	− 0.423	− 0.440	− 0.457	− 0.403	− 0.652	− 0.658	− 0.596	− 0.679	0.917	− 0.888	0.926	0.388	0.533	− 0.006	0.212	− 0.061	1

## Footnotes

1 Italy is territorially divided into 21 NUTS-2 (Nomenclature of Units for Territorial Statistics of the European Union) regions where 2 indicates basic regions for development policies and European funds management. In Italy, NUTS-2 regions correspond to the administrative regions, except for Trentino Alto Adige (two autonomous provinces of Bolzano and Trento).

2 The choice of weighting methods was limited to objective criteria and dependent on their compatibility with aggregation methods (OECD, 2008, p. 31). First, weighting techniques derived from participatory methods (i.e. budget allocation processes, analytic hierarchy processes, and conjoint analysis) were excluded because not free of subjectivity, even if based on expert assessments. Second, in the context of analytical approaches, the BoD (Benefit of Doubt) method was also omitted. While satisfying the compatibility criterion for linear aggregation, the BoD weights are country specific (in this paper, region specific) and as such more difficult to interpret and compare with those from PCA. Using the BoD method, different sets of weights may lead to the selection of different regions and the benchmark would generally be region-dependent; therefore, there would be no unique benchmark, as long as no one region has the highest score in all elementary indicators (OECD, 2008, p. 93). This makes the comparison (and interpretation) of the output (scores, rankings) of the composite indicators obtained from different weighting methods hard to do.
